# Mixed Sessions

**DOI:** 10.1007/978-3-030-44914-8_26

**Published:** 2020-04-18

**Authors:** Vasco T. Vasconcelos, Filipe Casal, Bernardo Almeida, Andreia Mordido

**Affiliations:** ETH Zurich, Zurich, Switzerland; LASIGE, Faculdade de Ciências, Universidade de Lisboa, Lisbon, Portugal

**Keywords:** Type Systems, Session Types, Mixed Choice

## Abstract

Session types describe patterns of interaction on communicating channels. Traditional session types include a form of choice whereby servers offer a collection of options, of which each client picks exactly one. This sort of choice constitutes a particular case of separated choice: offering on one side, selecting on the other. We introduce mixed choices in the context of session types and argue that they increase the flexibility of program development at the same time that they reduce the number of synchronisation primitives to exactly one. We present a type system incorporating subtyping and prove preservation and absence of runtime errors for well-typed processes. We further show that classical (conventional) sessions can be faithfully and tightly embedded in mixed choices. Finally, we discuss algorithmic type checking and a runtime system built on top of a conventional (choice-less) message-passing architecture.

## 1 Introduction

Session types provide for describing series of continuous interactions on communication channels [16,19,43,45,49]. When used in type systems for programming languages, session type systems statically verify that programs follow protocols, and hence that they do not engage in communication mismatches.

In order to motivate mixed sessions, suppose that we want to describe a process that asks for a fixed but unbounded number of integer values from some producer. The consumer may be in two states: happy with the values received so far, or ready to ask the producer for a new value. In the former case it must notify the producer so that this may stop sending numbers. In the latter case, the client must ask the producer for another integer, after which it "goes back to the beginning". Using classical sessions, and looking from the consumer side, the communication channel can be described by a (recursive) session type T of the form

$$\oplus\{\mathsf{enough}:\mathbf{end},\ \mathsf{more}:\ ?\mathbf{int}.\mathsf{T}\}$$

where $\oplus$ denotes internal choice (the consumer decides), the two branches in the choice are labelled with enough and more, type **end** denotes a channel on which no further interaction is possible, and ?**int** denotes the reception of an integer value. Reception is a prefix to a type, the continuation is T (in this case the "goes back to the beginning" part). The code for the consumer (and the producer as well) is unnecessarily complex, featuring parts that exchange messages in both directions: enough and more selections from the consumer to the producer, and **int** messages from the producer to the consumer. In particular, the consumer must first select option enough (outgoing) and then receive an integer (incoming).

Using mixed sessions one can *invert the direction* of the more selection and write the type of the channel (again as seen from the side of the consumer) as

$\oplus\{$enough!**unit**.**end**, more?**int**.T$\}$

The changes seem merely cosmetic, but label/polarity pairs (polarity is ! or ?) are now indivisible and constitute the keys of the choice type when seen as a map. The integer value is piggybacked on top of selection more. As a result, the classical session primitive operations: selection and branching (that is, internal and external choice) and communication (output and input) become one only: mixed session. The producer can be safely written as p (enough?z. **0** + more!n. produce!(p, n+1))

offering a choice on channel end p featuring mixed branches with labels enough? and more!, where **0** denotes the terminated process and produce(p, n+1) a recursive call to the producer. The example is further developed in Section 2.

Mixed sessions build on Vasconcelos presentation of session types which we call *classical sessions* [43], by adapting choice and input/output as needed, but keeping everything else unchanged as much as possible. The result is a language with

- a single synchronisation/communication primitive: mixed choice on a given channel that
- allows for duplicated labels in choice processes, leading to non-determinism in a pure linear setting, and
- replicated output processes arising naturally from replicated mixed choices, and that
- enjoys preservation and absence of runtime errors for typable processes, and
- provides for embedding classical sessions in a tight type and operational correspondence.

The rest of the paper is organised as follows: the next section shows mixed sessions in action; Section 3 introduces the technical development of the language, and Section 4 proves the main results (preservation and absence of runtime errors for typable processes). Then Section 5 presents the embedding and the correspondence proofs, Section 6 discusses implementation details, and Section 7 explores related work. Section 8 concludes the paper.

## 2 There is Room for Mixed Sessions

This section introduces the main ideas of mixed sessions via examples. We address *mixed choices*, *duplicated labels in choices*, and *unrestricted output*, in this order.

## 2.1 Mixed Choices

Consider the producer-consumer problem where the producer produces only insofar as so requested by the consumer. Here is the code for a producer that writes on channel end x numbers starting from n.

```
def produce (x, n) =
  lin x (enough?z. 0 +
         more!n. produce!(x, n+1)
  )
```

Syntax qx(M+N) introduces a choice between M and N on channel end x. Qualifier q is either **un** or **lin** and controls whether the process is persistent (remains after reduction) or is ephemeral (is consumed in the reduction process). Each branch in a choice is composed of a label (enough or more), a polarity mark (input ? or output !), a variable or a value (z or n), and a continuation process (after the dot). The terminated process is represented by **0**; notation **def** introduces a recursive process. The **def** syntax and its encoding in the base language is from the Pict programming language [36] and taken up by Sepi [12].

A consumer that requests n integer values on channel end y can be written as follows, where () represents the only value of type **unit**.

```
def consume (y, n) =
  if n == 0
  then lin y (enough!(). 0)
  else lin y (more?z. consume!(x, n-1))
```

Suppose that x and y are two ends of the same channel. When choices on x and on y get together, a pair of matching label-polarities pairs is selected and a value transmitted from the output continuation to the input continuation.

Types for the two channel ends ensure that choice synchronisation succeeds. The type of x is **rec** a. **lin** &{enough?**unit**.**end**, more!**int**.a} where the qualifier **lin** says that the channel end must be used in exactly one process, & denotes external choice, and each branch is composed of a label, a polarity mark, the type of the communication, and that of the continuation. The type **end** states that no further interaction is possible at the channel and **rec** introduces a recursive type. The type of y is obtained from that of x by inverting views (⊕ and &) and polarities (! and ?), yielding **rec** b. **lin** ⊕{enough!**unit**.**end**, more?**int**.b}. The choice at x in the produce process contains all branches in the type and so we select an external choice view & for x. The choices at y contain only part of the branches, hence the internal choice view ⊕. This type discipline ensures that processes do not engage in runtime errors when trying to find a match for two choices at the two ends of a given channel.

A few type and process abbreviations simplify coding: i) the **lin** qualifier can be omitted, ii) the terminated process **0** together with the trailing dot can be omitted; iii) the terminated type **end** together with the trailing dot can be omitted; and iv) we introduce wildcards (_) in variable binding positions (in input branches).

## 2.2 Duplicated Labels in Choices for Types and for Processes

Classical session types require distinct identifiers to label distinct branches. Mixed sessions relax this restriction by allowing duplicated labels whenever paired with distinct polarities. The next example describes two processes—countDown and collect—that bidirectionally exchange a fixed number of msg-labelled messages. The number of messages that flow in each direction is not fixed a priori, but instead decided by the non-deterministic operational semantics. The type that describes the channel, as seen by process countDown, is **rec** a.⊕{msg!**unit**.a, msg?**unit**.a, done!**unit**}, where one can see the msg label in two distinct branches, but with different polarities.

Process countDown features a parameter n that controls the number of messages exchanged (sent or received). The end of the interaction (when n reaches 0) is signalled by a done message.

```
countDown : (rec a.⊕{msg!unit.a, msg?unit.a, done!unit}, int)
def countDown (x, n) =
  if n == 0
  then x (done!())
  else x (msg!(). countDown!(x, n−1) +
          msg?_. countDown!(x, n−1))
```

Process collect sees the channel from the dual viewpoint, obtained by exchanging ? with ! and ⊕ with &. Parameter n in this case denotes the number of messages received. When done, the process writes the result on channel end r, global to the collect process.

```
collect : (rec b.&{msg!unit.b, msg?unit.b, done?unit}, int)
def collect (y, n) =
  y (msg!(). collect!(y, n+1) +
     msg?_. collect!(y, n) +
     done?_. r (result!n))
```

Mixed sessions allow for duplicated message-polarity pairs permitting a new form of non-determinism that uses exclusively linear channels. A process of the form $(\nu \mathsf{xy})\mathsf{P}$ declares a channel with end points x and y to be used in process P. The process

```
(νxy)(
  x (msg!()) |
  y (msg?_. z (m!true) + msg?_. z (m!false))
)
```

featuring two linear choices may reduce to z (m!**true**) or to z (m!**false**). Non-determinism in the $\pi$-calculus without choice (that of *Functions as Processes* [27,29] for example) can only be achieved by introducing race conditions on un channels. For example, the $\pi$-calculus process

```
(νxy)(x!() | y?_.z!true | y?_.z!false))
```

reduces either to $(\mathsf{z!}\textbf{true} \mid (\nu \mathsf{xy})\mathsf{y?\_.z!}\textbf{false}))$ or to $(\mathsf{z!}\textbf{false} \mid (\nu \mathsf{xy})\mathsf{y?\_.z!}\textbf{true}))$, leaving for the runtime the garbage collection of the inert residuals. Also note that in this case, channel y cannot remain linear.

Duplicated message-polarities in choices lead to elegant and concise code. A random number generator with a given number n of bits can be written with two processes. The first process sends n messages on channel end x. The contents of the messages are irrelevant (we use value () of type **unit**); what is important is that n more messages are sent, followed by a done message, followed by silence.

```
write : (rec a.⊕{done!unit, more!unit.a}, int)
def write (x, n) =
  if n == 0
  then x(done!())
  else x(more!(). write!(x, n−1))
```

The reader process reads the more messages in two distinct branches and interprets messages received on one branch as bit 0, and on the other as 1. Upon the reception of a done message, the accumulated random number is conveyed on channel end r, a variable global to the read process.

```
read : (rec b.&{done?unit, more?unit.b}, int)
def read (y, n) =
  y (done?_. r (result!n) +
     more?_. read!(y, 2*n) +
     more?_. read!(y, 2*n+1)
  )
```

Notice that mixed sessions allow duplicated label-polarity pairs in processes but not in types. This point is further discussed in Section 3. Also note that duplicated message labels could be easily added to traditional session types.

### 2.3 Unrestricted Output

Mixed sessions allow for replicated output processes. The original version of the $\pi$-calculus [30,31] features recursion on arbitrary processes. Subsequent versions [29] introduce replication but restricted to input processes. When compared to languages with unrestricted input only, unrestricted output allows for more concise programs and fewer message exchanges for the same effect. Here is a process (call it $P$) containing a pair of processes that exchange msg-labelled messages ad-aeternum,

```
(νxy)(un y (msg!()) | un x (msg?_))
```

where x is of type **rec** a.**un** &{msg?**unit**.a}. The un prefix denotes replication: an un choice survives reduction. Because none of the two sub-processes features a continuation $P$ reduces to $P$ in one step. The behaviour of **un** y (msg!()) can be mimicked by a process without output replication, namely,

```
(νwz) w (ℓ!()) | un z (ℓ?_. y (msg!(). w (ℓ!())))
```

| | | | |
|---|---|---|---|
| $v$ | $::=$ | | Values: |
| | | $x$ | variable |
| | | $\mathsf{true} \mid \mathsf{false}$ | boolean values |
| | | $()$ | unit value |
| $P$ | $::=$ | | Processes: |
| | | $qx \sum_{i \in I} M_i$ | choice |
| | | $P \mid P$ | parallel composition |
| | | $(\nu xx)P$ | scope restriction |
| | | $\mathsf{if}\ v\ \mathsf{then}\ P\ \mathsf{else}\ P$ | conditional |
| | | $\mathbf{0}$ | inaction |
| $M$ | $::=$ | | Branches: |
| | | $l^\star v.P$ | branch |
| $\star$ | $::=$ | | Polarities: |
| | | $! \mid ?$ | out and in |
| $q$ | $::=$ | | Qualifiers: |
| | | $\mathsf{lin} \mid \mathsf{un}$ | linear and unrestricted |

Fig. 1: The syntax of processes

Even if unrestricted output can be simulated with unrestricted input, the encoding requires one extra channel ($\mathsf{wz}$) and an extra message exchange (on channel $\mathsf{wz}$) in order to reestablish the output on channel end $\mathsf{y}$.

It is a fact that unrestricted output can be added to any flavour of the $\pi$-calculus (session-typed or not). In the case of mixed sessions it arises naturally: there is only one communication primitive—choice—and this can be classified as $\mathsf{lin}$ or $\mathsf{un}$. If an $\mathsf{un}$-choice happens to behave in "output mode", then we have an $\mathsf{un}$-output. It is not obvious how to design the language of mixed choices without allowing unrestricted output, while still allowing unrestricted input (which is mandatory for unbounded behaviour).

## 3 The Syntax and Semantics of Mixed Sessions

This section introduces the syntax and the semantics of mixed sessions. Inspired in Vasconcelos' formulation of session types for the $\pi$-calculus [43,45], mixed sessions replace input and output, selection and branching (internal and external choice), with a single construct which we call *choice*.

### 3.1 Syntax

Figure 1 presents the syntax of values and processes. Let $x, y, z$ range over a (countable) set of *variables*, and let $l$ range over a set of *labels*. Metavariable $v$ ranges over *values*. Following the tradition of the $\pi$-calculus, set up by Milner et al. [30,31], variables are used both as placeholders for incoming values in communication and for channels. Linearity constraints, central to session types but absent in the $\pi$-calculus, dictate that the two ends of a channel must be syntactically distinguished; we use one variable for each end [43]. Different primitive values can be used. Here, we pick the boolean values (so that we may have a conditional process), and unit that plays its role in the embedding of classical session types (Section 5).

Metavariables $P$ and $Q$ range over processes. Choices are processes of the form $qx \sum_{i\in I} M_i$ offering a choice of $M_i$ alternatives on channel end $x$. Qualifier $q$ describes how choice behaves with respect to reduction. If $q$ is $\mathsf{lin}$, then the choice is consumed in reduction, otherwise $q$ must be $\mathsf{un}$, and in this case the choice persists after reduction. The type system in Figure 8 rejects nullary (empty) choices. There are two forms of branches: output $l^! v.P$ and input $l^? x.P$. An output branch sends value $v$ and continues as $P$. An input branch receives a value and continues as $P$ with the value replacing variable $x$. The type system in Figure 8 makes sure that value $v$ in $l^? v.P$ is a variable.

The remaining process constructors are standard in the $\pi$-calculus. Processes of the form $P \mid Q$ denote the parallel composition of processes $P$ and $Q$. Scope restriction $(\nu xy)P$ binds together the two channel ends $x$ and $y$ of a same channel in process $P$. The conditional process $\mathsf{if}\ v\ \mathsf{then}\ P\ \mathsf{else}\ Q$ behaves as process $P$ if $v$ is $\mathsf{true}$ and as process $Q$ otherwise. Since we do not have nullary choices, we include $\mathbf{0}$—called inaction—as primitive to denote the terminated process.

### 3.2 Operational Semantics

The variable bindings in the language are as follows: variables $x$ and $y$ are bound in $P$, in a process of the form $(\nu xy)P$; variable $x$ is bound in $P$ in a choice of the form $l^? x.P$. The sets of bound and free variables, as well as substitution, $P[v/x]$, are defined accordingly. We work up to alpha-conversion and follow Barendregt's variable convention, whereby all variables in binding occurrences in any mathematical context are pairwise distinct and distinct from the free variables [2].

Figure 2 summarises the operational semantics of mixed sessions. Following the tradition of the $\pi$-calculus, a binary relation on processes—*structural congruence*—rearranges processes when preparing for reduction. Such an arrangement reduces the number of rules included in the operational semantics. Structural congruence was introduced by Milner [27,29]. It is defined as the least congruence relation closed under the axioms in Figure 2. The first three rules state that parallel composition is commutative, associative, and takes inaction as the neutral element. The fourth rule is commonly known as scope extrusion [30,31] and allows extending the scope of channel ends $x, y$ to process $Q$. The side-condition

*Structural congruence,* $P \equiv P$

$$P \mid Q \equiv Q \mid P \qquad (P \mid Q) \mid R \equiv P \mid (Q \mid R) \qquad P \mid \mathbf{0} \equiv P$$
$$(\nu xy)P \mid Q \equiv (\nu xy)(P \mid Q) \qquad (\nu xy)\mathbf{0} \equiv \mathbf{0} \qquad (\nu wx)(\nu yz)P \equiv (\nu yz)(\nu wx)P$$

*Reduction,* $P \to P$

$$\mathsf{if\ true\ then}\ P\ \mathsf{else}\ Q \to P \qquad \mathsf{if\ false\ then}\ P\ \mathsf{else}\ Q \to Q \qquad \text{[R-IfT] [R-IfF]}$$

$$(\nu xy)(\mathsf{lin}x(M + l^! v.P + M') \mid \mathsf{lin}y(N + l^? z.Q + N') \mid R) \to (\nu xy)(P \mid Q[v/z] \mid R) \qquad \text{[R-LinLin]}$$

$$(\nu xy)(\mathsf{lin}x(M + l^! v.P + M') \mid \mathsf{un}y(N + l^? z.Q + N') \mid R) \to (\nu xy)(P \mid Q[v/z] \mid \mathsf{un}y(N + l^? z.Q + N') \mid R) \qquad \text{[R-LinUn]}$$

$$(\nu xy)(\mathsf{un}x(M + l^! v.P + M') \mid \mathsf{lin}y(N + l^? z.Q + N') \mid R) \to (\nu xy)(P \mid Q[v/z] \mid \mathsf{un}x(M + l^! v.P + M') \mid R) \qquad \text{[R-UnLin]}$$

$$(\nu xy)(\mathsf{un}x(M + l^! v.P + M') \mid \mathsf{un}y(N + l^? z.Q + N') \mid R) \to (\nu xy)(P \mid Q[v/z] \mid \mathsf{un}x(M + l^! v.P + M') \mid \mathsf{un}y(N + l^? z.Q + N') \mid R) \qquad \text{[R-UnUn]}$$

$$\frac{P \to Q}{(\nu xy)P \to (\nu xy)Q} \qquad \frac{P \to Q}{P \mid R \to Q \mid R} \qquad \frac{P \equiv P' \quad P' \to Q' \quad Q' \equiv Q}{P \to Q}$$
$$\text{[R-Res] [R-Par] [R-Struct]}$$

Fig. 2: Operational semantics

"$x$ and $y$ not free in $Q$" is redundant in face of the Barendregt convention. The fifth rule allows collecting channel bindings no longer in use, and the last rule allows for rearranging the order of channel bindings in a process.

Reduction includes six axioms, two for the destruction of boolean values (via a conditional process), and four for communication. The axioms for communication take processes of a similar nature. The scope restriction $(\nu xy)$ identifies the two ends of the channel engaged in communication. Under the scope of the channel one finds three processes: the first contains an output process on channel end $x$, the second contains an input process on channel end $y$, and the third $(R)$ is an arbitrary process that may contain other references to $x$ and $y$ (the witness process). Communication proceeds by identifying a pair of compatible branches, namely $l^! v.P$ and $l^? z.Q$. The result contains the continuation process $P$ and the continuation process $Q$ with occurrences of the bound variable $z$ replaced by value $v$ (together with the witness process). The four axioms differ in the treatment of the process qualifiers: $\mathsf{lin}$ (ephemeral) and $\mathsf{un}$ (persistent). Ephemeral processes are consumed in reduction, persistent processes remain in the contractum.

Choices apart, rules [R-LinLin] and [R-LinUn] are already present in the works of Milner and Vasconcelos [29,43]. Rules [R-UnLin] and [R-LinLin] are absent on the grounds of economy: replicated output can be simulated with a new channel and a replicated in input. In mixed choices these rules cannot be

| | | |
|---|---|---|
| $T$ ::= | | Types: |
| | $q\sharp\{U_i\}_{i\in I}$ | choice |
| | $\mathsf{end}$ | termination |
| | $\mathsf{unit} \mid \mathsf{bool}$ | unit and boolean |
| | $\mu a.T$ | recursive type |
| | $a$ | type variable |
| $U$ ::= | | Branches: |
| | $l^\star T.T$ | branch |
| $\sharp$ ::= | | Views: |
| | $\oplus \mid \&$ | internal and external |
| $\Gamma$ ::= | | Contexts: |
| | $\cdot$ | empty |
| | $\Gamma, x\colon T$ | entry |

Fig. 3: The syntax of types omitted for there is no distinction between input and output: choice is the only (symmetrical) communication primitive.

We have designed mixed choices in such a way that labels may be duplicated in choices; more: label-polarity pairs may be also be duplicated. This allows for non-determinism in a linear context. For example, process

$$(\nu xy)(\mathsf{lin}\,x(l^!\mathsf{true}.\mathbf{0} + l^!\mathsf{false}.\mathbf{0}) \mid \mathsf{lin}\,y(l^?z.\mathsf{lin}\,w(m^!z.\mathbf{0})))$$

reduces in one step to either $\mathsf{lin}\,w(m^!\mathsf{true}.\mathbf{0})$ or $\mathsf{lin}\,w(m^!\mathsf{false}.\mathbf{0})$.

The examples in Section 2 take advantage of a $\mathsf{def}$ notation, a derived process construct inspired in the SePi [12] and the Pict languages [36]. A process of the form $\mathsf{def}\ x(z) = P\ \mathsf{in}\ Q$ is understood as

$$(\nu xy)(\mathsf{un}\,y(\ell^?z.P) \mid Q))$$

and calls to the recursive procedure, of the form $x!v$, are interpreted as $\mathsf{lin}\,x(\ell^!v)$, for $\ell$ an arbitrarily chosen label. The derived syntax hides channel end $y$ and simplifies the syntax of calls to the procedure. Procedures with more than one parameter require tuple passing, a notion that is not primitive to mixed sessions. Fortunately, tuple passing is easy to encode; see Vasconcelos[43].

### 3.3 Typing

Figure 3 summarises the syntax of types. We rely on an extra set, that of *type variables*, $a, b, \ldots$ Types describe values, including boolean and unit values, and

*Branch subtyping*, $U <: U$

$$\frac{S_2 <: S_1 \quad T_1 <: T_2}{l^! S_1.T_1 <: l^! S_2.T_2} \qquad \frac{S_1 <: S_2 \quad T_1 <: T_2}{l^? S_1.T_1 <: l^? S_2.T_2}$$

*Subtyping*, $T <: T$

$$\frac{}{\mathsf{end} <: \mathsf{end}} \qquad \frac{}{\mathsf{unit} <: \mathsf{unit}} \qquad \frac{}{\mathsf{bool} <: \mathsf{bool}} \qquad \frac{S[\mu a.S/a] <: T}{\mu a.S <: T} \qquad \frac{S <: T[\mu a.T/a]}{S <: \mu a.T}$$

$$\frac{J \subseteq I \qquad U_j <: V_j}{q\oplus\{U_i\}_{i\in I} <: q\oplus\{V_j\}_{j\in J}} \qquad \frac{I \subseteq J \qquad U_i <: V_i}{q\&\{U_i\}_{i\in I} <: q\&\{V_j\}_{j\in J}}$$

Fig. 4: Coinductive subtyping rules channel ends. A type of the form $q\sharp\{U_i\}_{i\in I}$ denotes a channel end. Qualifier $q$ states the number of processes that may contain references to the channel end: exactly one for lin, zero or more for un. View $\sharp$ distinguishes external ($\oplus$) from internal ($\&$) choice. This distinction is not present in processes but is of paramount importance for typing purposes, as we shall see. The branches are either of output—$l^! S.T$—or of input—$l^? S.T$—nature. In either case, $S$ denotes the object of communication and $T$ describes the subsequent behaviour of the channel end. Type end denotes the channel end on which no more interaction is possible. Types $\mu a.T$ and $a$ cater for recursive types.

Types are subject to a few syntactic restrictions: i) choices must have at least one branch; ii) label-polarity pairs—$l^\star$—are pairwise distinct in the branches of a choice type (unlike in processes); iii) recursive types are assumed contractive (that is, containing no subterm of the form $\mu a_1 \dots \mu a_n.a_1$). New variables, new bindings: type variable $a$ is bound in $T$ in type $\mu a.T$. Again the definitions of bound and free names as well as that of substitution—$S[T/a]$—are defined accordingly.

Mixed sessions come equipped with a notion of subtyping. Figure 4 introduces the rules that allow determining whether a given type is subtype of another. The rules must be read coinductively. Base types (end, unit, bool) are subtypes to themselves. The rules for recursive types are standard. Subtyping behaves differently in presence of external or internal choice. For external choice we require the branches in the subtype to contain those in the supertype: exercising less options cannot cause difficulties on the receiving side. For internal choice we require the opposite: here offering more choices can not cause runtime errors. For branches we distinguish output from input: output is contravariant on the contents of the message, input is covariant. In either case, the continuation is covariant. Choices, input/output, and recursive types receive no different treatment than those in classical sessions [15]. We can easily show that the $<:$ relation is a preorder. Notation $S \equiv T$ abbreviates $S <: T$ and $T <: S$.

Duality is a notion central to session types. In order for channel communication to proceed smoothly, the two channel ends must be compatible: if one end says input, the other must say output; if one end says external choice, the

*Polarity duality and view duality,* $\sharp \perp \sharp$ and $\star \perp \star$

$$! \perp ? \qquad ? \perp ! \qquad\qquad \oplus \perp \& \qquad \& \perp \oplus$$

*Type duality,* $T \perp T$

$$\frac{}{\mathsf{end} \perp \mathsf{end}} \qquad \frac{\sharp \perp \flat \qquad \star_i \perp \bullet_i \qquad S_i \equiv S_i' \qquad T_i \perp T_i'}{q\sharp\{l_i^\star S_i.T_i\}_{i\in I} \perp q\flat\{l_i^\bullet S_i'.T_i'\}_{i\in I}}$$

$$\frac{S[\mu a.S/a] \perp T}{\mu a.S \perp T} \qquad \frac{S \perp T[\mu a.T/a]}{S \perp \mu a.T}$$

Fig. 5: Coinductive type duality rules un *and* lin *predicates,* $\mathsf{un}(T)$, $\mathsf{lin}(T)$

$$\mathsf{un}(\mathsf{end}) \quad \mathsf{un}(\mathsf{unit}) \quad \mathsf{un}(\mathsf{bool}) \quad \mathsf{un}(\mathsf{un}\sharp\{U_i\}) \quad \frac{\mathsf{un}(T)}{\mathsf{un}(\mu a.T)} \qquad\qquad \frac{}{\mathsf{lin}(T)}$$

Fig. 6: The un and lin predicates on types other must say internal choice. In presence of recursive types, the problem of building the dual of a given type has been elusive, as works by Bernardi and Hennessy, Bono and Padovani, Lindley and Morris show [5,7,25]. Here we eschew the problem by working with a duality relation, as in Gay and Hole [15].

The rules in Figure 5 define what we mean for two types to be dual. This is the coinductive definition of Gay and Hole in rule format (and adapted to choice). Duality is defined for session types only. Type end is the dual of itself. The rule for choice types requires dual views (& is the dual of $\oplus$, and vice-versa) and dual polarities (? is the dual of !, and vice-versa). Furthermore, the objects of communications must be equivalent ($S_i \equiv S_i'$) and the continuations must be dual again ($T_i \perp T_i'$). The rules in the second line handle recursion in the exact same way as in type equivalence. As an example, we can easily show that

$$\mu a.\mathsf{lin} \oplus \{l^?\mathsf{bool}.\mathsf{lin}\&\{m^!\mathsf{unit}.a\}\} \perp \mathsf{lin}\&\{l^!\mathsf{bool}.\mu b.\mathsf{lin}\oplus\{m^?\mathsf{unit}.\mathsf{lin}\&\{l^!\mathsf{bool}.b\}\}\}$$

It can be shown that $\perp$ is an involution, that is, if $R \perp S$ and $S \perp T$, then $R \equiv T$.

The meaning of the un and lin predicates are defined by the rules in Figure 6. Basic types—unit, bool, end—are unrestricted; un-annotated choices are unrestricted; $\mu a.T$ is unrestricted if $T$ is. Contractivity ensures that the predicate is total. All types are lin, meaning that both lin and non-lin types may be used in linear contexts.

Before presenting the type system, we need to introduce two notions that manipulate typing contexts. The rules in Figure 7 define the meaning of *context split* and *context update*. These two relations are taken verbatim from Vasconcelos [43]; context split is originally from Walker [48] (cf. Kobayashi et al. [22,23]). Context split is used when type checking processes with two sub-processes. In

*Context split*, $\Gamma = \Gamma \circ \Gamma$

$$\cdot = \cdot \circ \cdot \qquad \frac{\Gamma_1 \circ \Gamma_2 = \Gamma \qquad \mathsf{un}(T)}{\Gamma, x\colon T = (\Gamma_1, x\colon T) \circ (\Gamma_2, x\colon T)}$$

$$\frac{\Gamma = \Gamma_1 \circ \Gamma_2}{\Gamma, x\colon \mathsf{lin}\, p = (\Gamma_1, x\colon \mathsf{lin}\, p) \circ \Gamma_2} \qquad \frac{\Gamma = \Gamma_1 \circ \Gamma_2}{\Gamma, x\colon \mathsf{lin}\, p = \Gamma_1 \circ (\Gamma_2, x\colon \mathsf{lin}\, p)}$$

*Context update*, $\Gamma + x\colon T = \Gamma$

$$\frac{x\colon U \notin \Gamma}{\Gamma + x\colon T = \Gamma, x\colon T} \qquad \frac{\mathsf{un}(T) \qquad T \equiv U}{(\Gamma, x\colon T) + x\colon U = (\Gamma, x\colon T)}$$

Fig. 7: Inductive context split and context update rules this case we split the context in two, by copying unrestricted entries to both contexts and linear entries to one only. Context update is used to add to a given context an entry representing the continuation (after a choice operation) of a channel. If the variable in the entry is not in the context, then we add the entry to the context. Otherwise we require the entry to be present in the context and the type to be unrestricted.

The rules in Figure 8 introduce the typing system for mixed sessions. Here the $\mathsf{un}$ and $\mathsf{lin}$ predicates on types are pointwise extended to typing contexts. Notice that all contexts are linear and only some contexts are unrestricted. We require all instances of the axioms to be built from unrestricted contexts, thus ensuring that linear resources (channel ends) are fully consumed in typing derivations.

The typing rules for values should be straightforward: constants have their own types, the type for a variable is read from the context, and [T-Sub] is the subsumption rule, allowing a type to be replaced by a supertype.

The rules for branches—[T-Out] and [T-In]—follow those for output and input in classical session types. To type an output branch we split the context in two: one part for the value, the other for the continuation process. To type an input branch we add an entry with the bound variable $x$ to the context under which we type the continuation process. Rule [T-In] rejects branches of the form $l^?v.P$ when $v$ not a variable. The continuation type $T$ is not used in neither rule; instead it is incorporated in the type for the channel in $\Gamma$ (cf. rule [T-Choice] below).

The rules for inaction, parallel composition, and conditional are from Vasconcelos [43]. That for scope restriction is adapted from Gay and Hole [15]. Rule [T-Inact] follows the general pattern for axioms, requiring a $\mathsf{un}$ context. Rule [T-Par] splits the context in two, providing each subprocess with one part. Rule [T-If] splits the context and uses one part to type guard $v$. Because $v$ is unrestricted, we know that $\Gamma_1$ contains exactly the $\mathsf{un}$ entries in $\Gamma_1 \circ \Gamma_2$ and that $\Gamma_2$ is equal to $\Gamma_1 \circ \Gamma_2$. Context $\Gamma_2$ is used to type *both* branches of the conditional, for only one of them will ever execute. Rule [T-Res] introduces in the typing context entries for the two channel ends, $x$ and $y$, at dual types.

*Typing rules for values*, $\Gamma \vdash v \colon T$

$$\frac{\mathsf{un}(\Gamma)}{\Gamma \vdash () \colon \mathsf{unit}} \qquad \frac{\mathsf{un}(\Gamma)}{\Gamma \vdash \mathsf{true}, \mathsf{false} \colon \mathsf{bool}} \qquad \frac{\mathsf{un}(\Gamma_1, \Gamma_2)}{\Gamma_1, x \colon T, \Gamma_2 \vdash x \colon T} \qquad \frac{\Gamma \vdash v \colon S \quad S <: T}{\Gamma \vdash v \colon T}$$

[T-Unit] [T-True] [T-False] [T-Var] [T-Sub]

*Typing rules for branches*, $\Gamma \vdash M \colon U$

$$\frac{\Gamma_1 \vdash v \colon S \qquad \Gamma_2 \vdash P}{\Gamma_1 \circ \Gamma_2 \vdash l^! v.P \colon l^! S.T} \qquad \frac{\Gamma, x \colon S \vdash P}{\Gamma \vdash l^? x.P \colon l^? S.T}$$

[T-Out] [T-In]

*Typing rules for processes*, $\Gamma \vdash P$

$$\frac{\mathsf{un}(\Gamma)}{\Gamma \vdash \mathbf{0}} \qquad \frac{\Gamma_1 \vdash P \qquad \Gamma_2 \vdash Q}{\Gamma_1 \circ \Gamma_2 \vdash P \mid Q}$$

[T-Inact] [T-Par]

$$\frac{\Gamma_1 \vdash v \colon \mathsf{bool} \qquad \Gamma_2 \vdash P \qquad \Gamma_2 \vdash Q}{\Gamma_1 \circ \Gamma_2 \vdash \mathsf{if}\ v\ \mathsf{then}\ P\ \mathsf{else}\ Q} \qquad \frac{\Gamma, x \colon S, y \colon T \vdash P \qquad S \perp T}{\Gamma \vdash (\nu xy)P}$$

[T-If] [T-Res]

$$\frac{q_1(\Gamma_1 \circ \Gamma_2) \quad \Gamma_1 \vdash x \colon q_2 \sharp\{l_i^\star S_i.T_i\}_{i \in I} \quad \Gamma_2 + x \colon T_j \vdash l_j^\star v_j.P_j \colon l_j^\star S_j.T_j \quad \{l_j^\star\}_{j \in J} = \{l_i^\star\}_{i \in I}}{\Gamma_1 \circ \Gamma_2 \vdash q_1 x \sum_{j \in J} l_j^\star v_j.P_j}$$

[T-Choice]

Fig. 8: Inductive typing rules

The rule for choice is new. The incoming context is split in two: one for the subject $x$ of the choice, the other for the various branches in the choice. The qualifier of the process, $q_1$, dictates the nature of the incoming context: $\mathsf{un}$ or $\mathsf{lin}$. This allows for a linear choice to contain channels of an arbitrary nature, but limits unrestricted choices to unrestricted channels only (for one cannot predict how many times such choices will be exercised). The second premise extracts a type $q_2\sharp\{l_i^\star S_i.T_i\}$ for $x$. The third premise types each branch: type $S_j$ is used to type values $v_j$ in the branches and each type $T_j$ is used to type the corresponding continuation. The rule updates context $\Gamma_2$ with the continuation type of $x$: if $q_2$ is $\mathsf{lin}$, then $x$ is not in $\Gamma_2$ and the update operation simply adds the entry to the context. If, on the other hand, $q_2$ is $\mathsf{un}$, then $x$ is in $\Gamma_2$ and the context update operation (together with rule [T-Sub]) insists that type $T_j$ is a subtype of $\mathsf{un}\sharp\{l_j^\star S_j.T_j\}$, meaning that $T_j$ is a recursive type.

The last premise to rule [T-Choice] insists that the set of labels in the choice type coincides with that in the choice process. That does not mean that the label-polarity pairs are in a one-to-one correspondence: label-polarity pairs are pairwise distinct in types (see the syntactic restrictions in Section 3.3), but not in processes. For example, process $\mathsf{lin}x(l^? y.\mathbf{0} + l^? z.\mathbf{0})$ can be typed against context $x \colon \mathsf{lin}\oplus\{l^?\mathsf{bool}.\mathsf{end}\}$. From the fact that the two sets must coincide does not follow that the label-polarity pairs type in the context must coincide with those in the process. Taking advantage of subtyping, the above process can still be typed against context $x \colon \mathsf{lin}\oplus\{l^?\mathsf{bool}.\mathsf{end}, m^!\mathsf{unit}.\mathsf{end}\}$ because $\mathsf{lin}\oplus\{l^?\mathsf{bool}.\mathsf{end}, m^!\mathsf{unit}.\mathsf{end}\} <: \mathsf{lin}\oplus\{l^?\mathsf{bool}.\mathsf{end}\}$. The opposite phenomenon happens with external choice, where one may remove branches by virtue of subtyping.

We complete this section by discussing examples that illustrate options taken in the typing system (we postpone the formal justification to Section 4). Suppose we allow empty choices in the syntax of types. Then the process

$$(\nu xy)(x() \mid y())$$

would be typable by taking $x\colon \oplus(), y\colon \&()$, yet the process would not reduce. We could add an extra reduction rule for the effect

$$(\nu xy)(x() \mid y() \mid R) \;\rightarrow\; (\nu xy)R$$

which would satisfy preservation (Theorem 2). We decided not to include it in our reduction rules as we did not want the extra complexity. Including the rule also does not bring any apparent benefit.

The syntax of processes places no restrictions on the label-polarity pairs in choices; yet that of types does. What if we relax the restriction that label-polarities pairs in choice types must be pairwise distinct? Then process

$$(\nu xy)(x(l^{!}\mathsf{true} + l^{!}()) \mid y(l^{?}z.\mathsf{if}\ z\ \mathsf{then}\ \mathbf{0}\ \mathsf{else}\ \mathbf{0}))$$

could be typed under context $x\colon \&\{l^{!}\mathsf{bool}, l^{!}\mathsf{unit}\}, y\colon \oplus\{l^{?}\mathsf{bool}, l^{?}\mathsf{unit}\}$, yet the process might reduce to $\mathsf{if}\ ()\ \mathsf{then}\ \mathbf{0}\ \mathsf{else}\ \mathbf{0}$ which is a runtime error.

## 4 Well-typed Mixed Sessions Do Not Lead to Runtime Errors

This section introduces the main results of mixed choices: absence of runtime errors and preservation, both for well-typed processes.

We say that a process is a *runtime error* if it is structurally congruent to:

– a process of the form

$$(\nu x_1 y_1)\dots(\nu x_n y_n)(\nu xy)(qx\sum_{i\in I} l_i^{\star} v_i.P_i \mid q'y\sum_{j\in J} l_j^{\star} w_j.Q_j \mid R)$$

where $\{l_i^{\bullet}\}_{i\in I} \cap \{l_j^{\star}\}_{j\in J} = \emptyset$ with each $\bullet_i$ is obtained by dualising $\star_i$, or
– a process of the form $qz(M + l^{?}v.P + N)$ and $v$ is not a variable, or
– a process of the form $\mathsf{if}\ v\ \mathsf{then}\ P\ \mathsf{else}\ Q$ and $v$ is neither $\mathsf{true}$ nor $\mathsf{false}$.

Examples of processes which are runtime errors include:

$$(\nu xy)(\mathsf{lin}x(l^{!}\mathsf{true}.\mathbf{0}) \mid \mathsf{lin}y(l^{!}\mathsf{true}.\mathbf{0}))$$
$$(\nu xy)(\mathsf{un}x(l^{!}\mathsf{true}.\mathbf{0}) \mid \mathsf{lin}y(m^{?}z.\mathbf{0}))$$
$$\mathsf{un}x(l^{?}\mathsf{false}.\mathbf{0})$$
$$\mathsf{if}\ ()\ \mathsf{then}\ \mathbf{0}\ \mathsf{else}\ \mathbf{0}$$

Notice that processes of the form $(\nu xy)\mathsf{lin} x \sum_{i\in I} M_i$ cannot be classified as runtime errors for they may be typed. Just think of $(\nu xy)\mathsf{lin}x(l^? z.\mathsf{lin}y(l^!\mathsf{true}.\mathbf{0}))$, typable under the empty context. Unlike the interpretations of session types in linear logic by Caires, Pfenning and Wadler [8,14,46,47], typable mixed session processes can easily deadlock. Similarly, processes with more than one lin-choice on the same channel end can be typed. For example process $\mathsf{lin}x(l^!\mathsf{true}.\mathbf{0}) \mid \mathsf{lin}x(l^? z.\mathbf{0}))$ can be typed under context $x\colon \mu a.\mathsf{un} \oplus \{l^!\mathsf{unit}.a, l^?\mathsf{bool}.a\}$. Recall the relationship between qualifiers in processes $q_1$ and those in types $q_2$ in the discussion of the rules for choice in Section 3.

**Theorem 1 (Well-typed processes are not runtime errors).** *If $\cdot \vdash P$, then $P$ is not a runtime error.*

*Proof.* In view of a contradiction, assume that $\cdot \vdash P$ and that $P$ is

$$(\nu x_1 y_1)\dots(\nu x_n y_n)(q_1 x_n \sum_{i\in I} l_i^\star v_i.P_i \mid q_2 y_n \sum_{j\in J} l_j^\star w_j.Q_j \mid R)$$

and $\{l_i^\bullet\}_{i\in I} \cap \{l_j^\star\}_{j\in J} = \emptyset$ with $\star_i \perp \bullet_i$. From the typing derivation for $P$, using [T-Par] and [T-Res], we obtain a context $\Gamma = \Gamma_1 \circ \Gamma_2 \circ \Gamma_3 = x_1\colon T_1, y_1\colon S_1, \dots, x_n\colon T_n, y_n\colon S_n$, $T_i \perp S_i$ for all $i = 1,\dots,n$ and that $\Gamma_1 \vdash q_1 x_n \sum_{i\in I} l_i^\star v_i.P_i$ and $\Gamma_2 \vdash q_2 y_n \sum_{j\in J} l_j^\star w_j.Q_j$ and $\Gamma_3 \vdash R$. Without loss of generality, due to the fact that $x_n$ and $y_n$ have dual types and from the premises of rule [T-Choice], assume that $\Gamma_1' \vdash x_n\colon q_1' \& \{l_k^\star T_k'.T_k''\}_{k\in K}$ and $\Gamma_2' \vdash y_n\colon q_2' \oplus \{l_k^\bullet S_k'.S_k''\}_{k\in K}$, $\{l_i^\star\}_{i\in I} = \{l_k^\star\}_{k\in K}$ and $\{l_j^\star\}_{j\in J} \subseteq \{l_k^\bullet\}_{k\in K}$, with $\star_k \perp \bullet_k$. This also implies that $\{l_i^\bullet\}_{i\in I} = \{l_k^\bullet\}_{k\in K}$. Thus, a label $l_j^\star$ from $q_2 y_1 \sum_{j\in J} l_j^\star w_j.Q_j$ belongs to the set of labels $\{l_i^\bullet\}_{i\in I}$: $l_j^\star \in \{l_k^\bullet\}_{k\in K} = \{l_i^\bullet\}_{i\in I}$, contradicting $\{l_i^\bullet\}_{i\in I} \cap \{l_j^\star\}_{j\in J} = \emptyset$ with $\star_i \perp \bullet_i$

When $P$ is $qz(M + l^? v.P + N)$ and $v$ is not a variable, the contradiction is with rule [T-Out], which can only be applied when the value $v$ is a variable.

When $P$ is $\mathsf{if}\ v\ \mathsf{then}\ P\ \mathsf{else}\ Q$ and $v$ is not a boolean value, the contradiction immediately arises with rule [T-If]. □

In order to prepare for the preservation result we introduce a few lemmas.

**Lemma 1 (Unrestricted weakening).** *If $\Gamma \vdash P$ and $\mathsf{un}(T)$, then $\Gamma, x\colon T \vdash P$.*

*Proof.* The proof goes by mutual induction on the rules for branches and processes, but we first need to show the result for the value typing rules. We need to show that if $\Gamma \vdash v\colon S$ and $\mathsf{un}(R)$ then $\Gamma, x\colon R \vdash v\colon S$. This follows by a simple case inspection of the rules [T-Unit], [T-True],[T-False],[T-Var] taking into consideration that $\mathsf{un}(R)$. For the rule [T-Sub], use the induction hypothesis to obtain $\Gamma, x\colon R \vdash v\colon S$ and conclude, using [T-Sub], that $\Gamma, x\colon R \vdash v\colon T$.

For the branch and processes typing rules we detail the proof when the last rule is [T-Out]. Using the result for typing values, we obtain $\Gamma_1, x\colon R \vdash v\colon S$, and the induction hypothesis for processes leads to $\Gamma_2, x\colon R \vdash P$. Using the un context split property, taking into account that $\mathsf{un}(R)$, we conclude that $\Gamma_1 \circ \Gamma_2, x\colon R \vdash l^! v.P\colon l^! S.T$.

For the processes rule [T-INACT], the result is a simple consequence of $\mathsf{un}(T)$. For the other rules, the result follows by induction hypothesis in processes and branches rules, as well as using the value typing result. We detail the proof for rule [T-IF]. Using the typing values result, we know that $\Gamma_1, x\colon T \vdash x\colon \mathsf{bool}$. By induction hypothesis we also obtain that $\Gamma_2, x\colon T \vdash P$ and $\Gamma_2, x\colon T \vdash Q$. Using the un context split property, we conclude $\Gamma_1 \circ \Gamma_2, x\colon T \vdash \mathsf{if}\ v\ \mathsf{then}\ P\ \mathsf{else}\ Q$. □

**Lemma 2 (Preservation for $\equiv$).** *If $\Gamma \vdash P$ and $P \equiv Q$, then $\Gamma \vdash Q$.*

*Proof.* As in Vasconcelos [43, Lemma 7.4] since we share the structural congruence axioms. □

**Lemma 3 (Substitution).** *If $\Gamma_1 \vdash v\colon T$ and $\Gamma_2, x\colon T \vdash P$ and $\Gamma = \Gamma_1 \circ \Gamma_2$, then $\Gamma \vdash P[v/x]$.*

*Proof.* The proof follows by mutual induction on the rules for processes and branches. □

**Theorem 2 (Preservation).** *If $\Gamma \vdash P$ and $P \to Q$, then $\Gamma \vdash Q$.*

*Proof.* The proof is by rule induction on the reduction, making use of the weakening, substitution lemmas, and preservation for structural congruence. We sketch the cases for [R-LINLIN] and [R-LINUN].

When reduction ends with rule [R-LINLIN], we know that rule [T-RES] introduces $x\colon X, y\colon Y$ with $X \perp Y$ in the context $\Gamma$. From there, with applications of [T-PAR] and [T-CHOICE], $\Gamma = \Gamma_1 \circ \Gamma_2 \circ \Gamma_3$ and $\Gamma_1 \vdash \mathsf{lin}\, x(M + l^! v.P + M')$, $\Gamma_2 \vdash \mathsf{lin}\, y(N + l^? z.Q + N')$, $\Gamma_3 \vdash R$. Furthermore, $\Gamma_1 = \Gamma_1' \circ \Gamma_1''$ and $\mathsf{lin}(\Gamma_1)$, $\Gamma_1' \vdash x\colon \mathsf{lin} \oplus \{M, l^! S.T, M'\}$ and $\Gamma_1'', x\colon T \vdash l^! v.P\colon l^! S.T$. From the [T-OUT] rule, $\Gamma_v \vdash v\colon S$ and $\Gamma_4 \vdash P$. For the $y$ side, $\Gamma_2' \vdash y\colon \mathsf{lin}\&\{N, l^? U.V, N'\}$ and $\Gamma_2'', y\colon Y \vdash l^? z.Q\colon l^? U.V$. From the [T-IN] rule, $\Gamma_z, y\colon V, z\colon U \vdash Q$. We also have that $S \equiv U$ from the duality of $x$ and $y$. Using the substitution Lemma 3, $\Gamma_z, y\colon V, \Gamma_v \vdash Q[v/z]$. Using [T-PAR] with the remaining contexts and [T-RES] types the conclusion of [R-LINLIN].

When reduction ends with rule [R-LINUN], we know that rule [T-RES] introduces $x\colon X, y\colon Y$ with $X \perp Y$ in the context $\Gamma$. From there, with applications of [T-PAR] and [T-CHOICE], $\Gamma = \Gamma_1 \circ \Gamma_2 \circ \Gamma_3$ and $\Gamma_1 \vdash \mathsf{lin}\, x(M + l^! v.P + M')$, $\Gamma_2 \vdash \mathsf{un}\, y(N + l^? z.Q + N')$, $\Gamma_3 \vdash R$. Furthermore, $\Gamma_1 = \Gamma_1' \circ \Gamma_1''$ and $\mathsf{lin}(\Gamma_1)$, $\Gamma_1' \vdash x\colon \mathsf{un} \oplus \{M, l^! S.T, M'\}$. Here $x$ is un since $x$ and $y$ are dual. We also have $\Gamma_1'', x\colon T \vdash l^! v.P\colon l^! S.T$, from which follows $\Gamma_4 \vdash v\colon S$ and $\Gamma_5 \vdash P$ from rule [T-OUT]. For the $y$ side, $\Gamma_2' \vdash y\colon \mathsf{un}\&\{N, l^? U.V, N'\}$ and $\Gamma_2'', y\colon Y \vdash l^? z.Q\colon l^? U.V$ which has $\Gamma_6, y\colon V, z\colon U \vdash Q$ from [T-IN].

Types $S$ and $U$ are equivalent due to the duality of $x, y$ and so $\Gamma_6, y\colon V, z\colon S \vdash Q$. Using the substitution Lemma 3, $\Gamma_6 \circ \Gamma_4, y\colon V \vdash Q[v/z]$. From $\Gamma_5$ we also type the process $P$. Using [T-PAR] with the remaining contexts and [T-RES], types the conclusion of [R-UNLIN]. □

## 5 Classical Sessions Were Mixed All Along

This section introduces the syntax and semantics of classical session types and shows that the language of classical sessions can be embedded in that of mixed sessions.

The syntax and semantics of classical session types are in Figure 9; we follow Vasconcelos [43]. The syntax and the rules for the various judgements extend those of Figures 1 to 8, where we remove choice both from grammar productions (for processes and types) and from the various judgements (operational semantics, subtyping, duality, and typing). On what concerns the syntax of processes, the choice construct of Figure 1 is replaced by new process constructors: output, linear (lin) and replicated (un) input, selection (internal choice) and branching (external choice). The four reduction axioms in Figure 2 that pertain to choice ([R-LinLin], [R-LinUn], [R-UnLin], [R-UnUn]) are replaced by the three axioms in Figure 9. Rule [R-LinCom] describes the output against ephemeral-input interaction, rule [R-UnCom] the output against replicated-input interaction, and rule [R-Case] selects a label in the menu at the other channel end.

The syntax of types features new constructs—linear or unrestricted input and output, and linear or unrestricted external and internal choice—replacing the choice construct in Figure 3. The subtyping rules for the new type constructors are taken from Gay and Hole [15]. Type duality is such that the objects of communication must be equivalent and the continuations (both in communication and choice) must be dual again. We omit the dual rules for $q!S.S' \perp q?T.T'$ and $q\&\{l_i\colon S_i\}_{i\in I} \perp q\oplus\{l_i\colon T_i\}_{i\in I}$. The new duality rules are adapted from the co-inductive definition of Gay and Hole [15]. The un predicate on types insists on the idea that un-annotated types are unrestricted: $\mathsf{un}(\mathsf{un} \star S.T)$ and $\mathsf{un}(\mathsf{un}\sharp\{l_i\colon T_i\})$. The typing rule for choice in Figure 8 is replaced by the four rules in Figure 9; these are taken verbatim from Vasconcelos [43].

The embedding of classical session types in mixed sessions is defined in Figure 10. It consists of two maps, one for processes, the other for types. These maps act as homomorphisms on all process and type constructors not explicitly shown. For example $[\![P \mid Q]\!] = [\![P]\!] \mid [\![Q]\!]$. We distinguish one label, msg, and use it to encode input and output (both processes and types). Input and output processes are encoded in choices with one only msg-labelled branch. The output process is qualified as lin (it does not survive reduction) and the input process reads its qualifier $q$ from the incoming process. Choice processes in classical sessions are encoded in choices in mixed sessions. The value transmitted on the mixed session is irrelevant: we pick () of type unit for the output side, and a fresh variable $y_i$ on the input side. Both types are linear.

Input and output types are translated in choice types. For output we *arbitrarily* pick an external choice ($\oplus$), and conversely for the input. The label in the only branch is msg in order to match our pick for processes, and the qualifier is read from the incoming type. For classical choices, we read the qualifier and the view from the incoming type. The type of the communication in the branches of the mixed choice is unit, again so that it matches our pick for processes.

Typing correspondence says that the embedding preserves typability.

*Classical syntactic forms*

$$
\begin{array}{llr}
P & ::= \dots & \text{Processes:} \\
& x!v.P & \text{output} \\
& qx?x.P & \text{input} \\
& x \triangleleft l.P & \text{selection} \\
& x \triangleright \{l_i \colon P_i\}_{i \in I} & \text{branching} \\
T & ::= \dots & \text{Types:} \\
& q \star T.T & \text{communication} \\
& q\sharp\{l_i \colon T_i\}_{i \in I} & \text{choice}
\end{array}
$$

*Classical reduction rules*, $P \to P$, (plus [R-Res] [R-Par] [R-Struct] from Figure 2)

$$(\nu xy)(x!v.P \mid \mathsf{lin}\, y?z.Q \mid R) \to (\nu xy)(P \mid Q[v/z] \mid R) \quad \text{[R-LinCom]}$$

$$(\nu xy)(x!v.P \mid \mathsf{un}\, y?z.Q \mid R) \to (\nu xy)(P \mid Q[v/z] \mid \mathsf{un}\, y?z.Q \mid R) \quad \text{[R-UnCom]}$$

$$\frac{j \in I}{(\nu xy)(x \triangleleft l_j.P \mid y \triangleright \{l_i \colon Q_i\}_{i \in I} \mid R) \to (\nu xy)(P \mid Q_j \mid R)} \quad \text{[R-Case]}$$

*Classical subtyping rules*, $T <: T$

$$\frac{T <: S \qquad S' <: T'}{q!S.S' <: q!T.T'} \qquad \frac{S <: T \qquad S' <: T'}{q?S.S' <: q?T.T'}$$

$$\frac{J \subseteq I \qquad S_j <: T_j}{q\oplus\{l_i \colon S_i\}_{i \in I} <: q\oplus\{l_j \colon T_j\}_{j \in J}} \qquad \frac{I \subseteq J \qquad S_i <: T_i}{q\&\{l_i \colon S_i\}_{i \in I} <: q\&\{l_j \colon T_j\}_{j \in J}}$$

*Classical type duality rules*, $T \perp T$

$$\frac{S \equiv T \qquad S' \perp T'}{q?S.S' \perp q!T.T'} \qquad \frac{S_i \perp T_i}{q \oplus \{l_i \colon S_i\}_{i \in I} \perp q\&\{l_i \colon T_i\}_{i \in I}}$$

*Classical typing rules*, $\Gamma \vdash P$

$$\frac{\Gamma_1 \vdash x \colon q\,!T.U \qquad \Gamma_2 \vdash v \colon T \qquad \Gamma_3 + x \colon U \vdash P}{\Gamma_1 \circ \Gamma_2 \circ \Gamma_3 \vdash x!v.P} \quad \text{[T-TOut]}$$

$$\frac{q_1(\Gamma_1 \circ \Gamma_2) \qquad \Gamma_1 \vdash x \colon q_2?T.U \qquad (\Gamma_2 + x \colon U), y \colon T \vdash P}{\Gamma_1 \circ \Gamma_2 \vdash q_1 x?y.P} \quad \text{[T-TIn]}$$

$$\frac{\Gamma_1 \vdash x \colon q\&\{l_i \colon T_i\}_{i \in I} \qquad \Gamma_2 + x \colon T_i \vdash P_i \qquad \forall i \in I}{\Gamma_1 \circ \Gamma_2 \vdash x \triangleright \{l_i \colon P_i\}_{i \in I}} \quad \text{[T-Branch]}$$

$$\frac{\Gamma_1 \vdash x \colon q\oplus\{l_i \colon T_i\}_{i \in I} \qquad \Gamma_2 + x \colon T_j \vdash P \qquad j \in I}{\Gamma_1 \circ \Gamma_2 \vdash x \triangleleft l_j.P} \quad \text{[T-Sel]}$$

Fig. 9: Classical session types

**Theorem 3 (Typing correspondence).**

1. *If $\Gamma \vdash v \colon T$, then $[\![\Gamma]\!] \vdash v \colon [\![T]\!]$.*
2. *If $\Gamma \vdash P$, then $[\![\Gamma]\!] \vdash [\![P]\!]$.*

*Process translation*

$$\begin{aligned}
[\![x!v.P]\!] &= \mathsf{lin}\, x\{\mathsf{msg}^! v.[\![P]\!]\}\\
[\![qx?y.P]\!] &= q\, x\{\mathsf{msg}^? y.[\![P]\!]\}\\
[\![x \lhd l.P]\!] &= \mathsf{lin}\, x\{l^!().[\![P]\!]\}\\
[\![x \rhd \{l_i\colon P_i\}_{i\in I}]\!] &= \mathsf{lin}\, x\{l_i^? y_i.[\![P_i]\!]\}_{i\in I} \quad (y_i \notin \mathrm{fv}(P_i))
\end{aligned}$$

(Homomorphic for $\mathbf{0}$, $P \mid Q$, $(\nu xy)P$, and $\mathsf{if}\, v\, \mathsf{then}\, P\, \mathsf{else}\, Q$)
*Type translation*

$$\begin{aligned}
[\![q!S.T]\!] &= q{\oplus}\{\mathsf{msg}^! [\![S]\!].[\![T]\!]\}\\
[\![q?S.T]\!] &= q\&\{\mathsf{msg}^? [\![S]\!].[\![T]\!]\}\\
[\![q{\oplus}\{l_i\colon T_i\}_{i\in I}]\!] &= q{\oplus}\{l_i^! \mathsf{unit}.[\![T_i]\!]\}_{i\in I}\\
[\![q\&\{l_i\colon T_i\}_{i\in I}]\!] &= q\&\{l_i^? \mathsf{unit}.[\![T_i]\!]\}_{i\in I}
\end{aligned}$$

(Homomorphic for $\mathsf{end}$, $\mathsf{unit}$, $\mathsf{bool}$, $\mu a.T$, and $a$)

Fig. 10: Embedding classical session types

*Proof.* 1. A straightforward rule induction on the hypothesis.

2. By rule induction on the hypothesis. We sketch a few cases.

When the derivation ends with [T-TIn], we use item 1., induction, the fact that $q_1(\Gamma_1 \circ \Gamma_2)$ implies $q_1[\![\Gamma_1 \cdot \Gamma_2]\!]$, and that $(\Gamma_2 + x\colon T), y\colon T = (\Gamma_1, y\colon T) + x\colon S$ because $x$ and $y$ are distinct variables.

When the derivation ends with [T-Branch], we obtain $(\Gamma_2 + x\colon T_i), y_i\colon \mathsf{unit} \vdash [\![P_i]\!]$ from the induction hypothesis $\Gamma_2 + x\colon T_i \vdash [\![P_i]\!]$ using weakening (Lemma 1). □

We complete this section by proving that the classical-mixed translation meets Gorla's good encoding criteria [17]. The five criteria proposed by Gorla ensure that the encoding is meaningful. There are two syntactical and three semantics-related criteria.

Let $\mathcal{C}$ range over classical processes and $\mathcal{M}$ range over mixed choice processes. The map $[\![\cdot]\!] : \mathcal{C} \to \mathcal{M}$ described in Figure 10 is a translation from classical processes to mixed choice processes. To be in line with the criteria, we add the process $\checkmark$ representing a successfully terminating process to the syntax of both the source and the target languages. We denote by $\Rightarrow$ the reflexive and transitive closure of the reduction relations, $\to$, in both the source and target languages. Sometimes we use subscript $\mathcal{M}$ to denote the reduction of mixed choice processes and the subscript $\mathcal{C}$ for the reduction of classical processes, even though it should be clear from context.

We say that a process $P$ does not reduce, $P \not\to$, when it cannot make any reduction step. We say that a process *diverges*, $P \to^\omega$, when $P$ can do an infinite number of reductions. On the other hand, a process is *successful*, $P \Downarrow$, if $P$ reduces to a process in parallel with a success $\checkmark$, that is, $P \Rightarrow P' \mid \checkmark$. Gorla's criteria view calculi as triples $\langle \mathcal{P}, \rightarrow, \asymp \rangle$, where $\mathcal{P}$ is a set of processes, $\rightarrow$ a reduction relation (the operational semantics), and $\asymp$ is a behavioral equivalence on processes.

The behavioral equivalence $\asymp$ for mixed sessions we use coincides with structural congruence $\equiv$.

The first criterion states that the translation is compositional. For this purpose, we define a context $\mathcal{C}(\_{1}; \ldots ; \_{k})$ as a classical process with $k$ holes.

**Theorem 4 (Compositionality).** *The translation* $[\![\cdot]\!] : \mathcal{C} \longrightarrow \mathcal{M}$ *is* compositional*, i.e., for every k-ary operator* op *of* $\mathcal{M}$ *and for every subset* $N$ *of channel ends, there exists a k-ary context* $\mathcal{C}^{N}_{\mathrm{op}}(\_{1}; \ldots ; \_{k})$ *such that for all* $P_1, \ldots, P_k$ *with* $\cup_{i=1}^{k} \mathrm{fv}(P_i) = N$ *and* $[\![\mathrm{op}(P_1, \ldots, P_k)]\!] = \mathcal{C}^{N}_{\mathrm{op}}([\![P_1]\!]; \ldots ; [\![P_k]\!])$.

*Proof.* The translation of a process is defined in terms of the translation of their subterms, see Figure 10. □

Following the ideas from Peters et al. [34], the translation from mixed to classical sessions can be enriched with a *renaming policy* $\varphi_{[\![\ ]\!]}$, representing a map from channel ends to sequences of channel ends. The following theorem states that the proposed translation is name invariant.

**Theorem 5 (Name invariance).** *The translation* $[\![\cdot]\!] : \mathcal{C} \longrightarrow \mathcal{M}$ *is* name invariant*, i.e., for every classical process* $P$ *and substitution* $\sigma$*,*

$$[\![P\sigma]\!] \begin{cases} = [\![P]\!]\sigma' & \text{if } \sigma \text{ is injective} \\ \asymp [\![P]\!]\sigma' & \text{otherwise} \end{cases}$$

*where* $\sigma'$ *is such that* $\varphi_{[\![\ ]\!]}(\sigma(x)) = \sigma'(\varphi_{[\![\ ]\!]}(x))$*, for every channel end* $x$*.*

*Proof.* The translation transforms each channel end ($x$, in Figure 10) into itself. Thus, any substitution is preserved. See Figure 10. □

Operational correspondence states that the embedding preserves and reflects reduction. In our case the embedding is quite tight: one reduction step in classical sessions corresponds to one reduction step in mixed sessions. There is no runtime penalty in running classical sessions on a mixed sessions machine. Further notice that we do not rely on any equivalence relation on mixed sessions to establish the result: mixed-sessions images leave no "junk" in the process of simulating classical sessions.

**Theorem 6 (Operational correspondence).** *Let* $P, P'$ *be classical sessions processes and* $Q$ *a mixed sessions process.*

1. *If* $P \rightarrow P'$*, then* $[\![P]\!] \rightarrow [\![P']\!]$*.*
2. *If* $[\![P]\!] \rightarrow Q$*, then* $P \rightarrow P'$ *and* $[\![P']\!] = Q$*, for some* $P'$*.*

*Proof.* Straightforward rule induction on the hypotheses, relying on the fact that $[\![P]\!][v/x] = [\![P[v/x]]\!]$ and $x_i \notin \mathrm{fv}(P_i)$ in the translation of $x \triangleright \{l_i \colon P_i\}_{i \in I}$. □

The following theorems concern the finite and infinite behavior of classical session processes and their corresponding translations.

**Theorem 7 (Divergence Reflection).** *The translation* $[\![\cdot]\!] : \mathcal{C} \longrightarrow \mathcal{M}$ reflects divergence*, i.e., if* $[\![P]\!] \rightarrow^{\omega}_{\mathcal{M}}$ *then* $P \rightarrow^{\omega}_{\mathcal{C}}$ *for every process* $P \in \mathcal{C}$*.*

*Proof.* Corollary of Theorem 6. □

**Theorem 8 (Success Sensitivity).** *The translation* $[\![\cdot]\!] : \mathcal{C} \longrightarrow \mathcal{M}$ *is* success sensitive*, i.e.,* $P \Downarrow_{\mathcal{C}}$ *iff* $[\![P]\!] \Downarrow_{\mathcal{M}}$*, for every process* $P \in \mathcal{C}$*.*

*Proof.* Corollary of Theorem 6. □

## 6 What is in the Way of a Compiler?

This section discusses algorithmic type checking and the implementation of choice in message passing architectures.

We start with type checking and then move to the runtime system. Gay and Hole present an algorithmic subtyping system for classical sessions [15]. Algorithmic subtyping for mixed sessions can be obtained by adapting the rules in Figure 4 along the lines of Gay and Hole. [T-Sub] is the only non syntax-directed rule in Figure 8.We delete this rule and distribute subtype checking among all rules that use, in their premises, sequents $\Gamma \vdash v\colon T$, as usual. Most of the rules include a non-deterministic context split operation. Take rule [T-Par], for example. Rather than guessing the right split, we take the incoming context and give it all to process $P$, later reclaiming the unused part. This outgoing context is then passed to process $Q$. The outgoing context of the parallel composition $P \mid Q$ is that of $Q$. See, e.g., Vasconcelos or Walker for details [43,48]. Rule [T-Res] requires guessing the type of the two channel ends, so that one is dual to the other. Rather than guessing the type of channel end $x$, we require the help of the programmer by working with an explicitly typed syntax—$(\nu xy : T)P$—as in Franco and Vasconcelos [12,43], where $T$ refers to the type of channel end $x$. For the type of channel end $y$, rather than guessing, we build it from type $T$; cf. [4,5,7,25].

Running mixed sessions on a message passing architecture need not be an expensive operation. Take one of the communication axioms in Figure 2. We set up a broker process that receives the label-polarity pairs of both processes ($\{l_i^\star\}_{i\in I}$ and $\{l_j^\star\}_{j\in J}$), decides on a matching pair (guaranteed to exist for typed processes), and communicates the result back to the two processes. The processes then exchange the appropriate value, and proceed. If the broker is an independent process, then we exchange five messages per choice synchronisation. This *basic broker* is instantiated for two processes $P \triangleq \mathsf{lin}\, x(l_1^? z.P_1 + l_2^! v_2.P_1 + l_3^! v_3.P_3)$ and $Q \triangleq \mathsf{lin}\, y(l_1^! v_1.Q_1 + l_3^? w.Q_3)$ in Figure 11a.

We can do better by piggybacking the values in the output choices together with the label-polarities pairs. The broker passes its decision to the input side in the form of a triple label-polarity-value, yielding one less message exchanged, as showcased in Figure 11b.

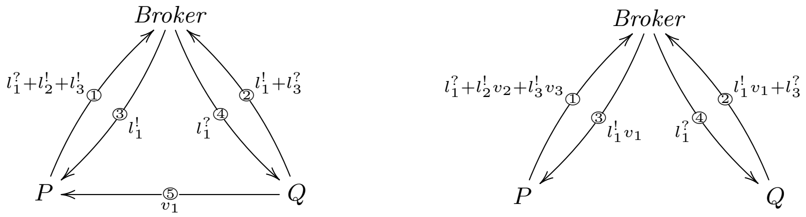

(a) Basic broker

(b) Values are piggybacked

Fig. 11: Broker is an independent process

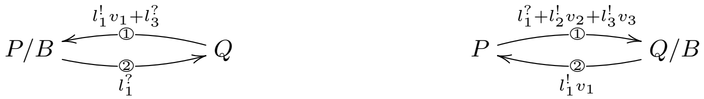

(a) $P$ is the broker

(b) $Q$ is the broker

Fig. 12: Broker is $P$ or $Q$

Finally, we observe that the broker need not be an independent process; it can be located at one of the choice processes. This reduces the number of messages down to two messages in the general case, as described in Figures 12a and 12b where either $P$ is the broker or $Q$ is the broker. Even if the value was already sent by $Q$ in the case that $P$ is the broker, $P$ must still let $Q$ know which choice was taken, so that $Q$ may proceed with the appropriate branch.

However, in particular cases one message may be enough. Take, for instance a process $P \triangleq \mathsf{un}\, x(l_1^! v_1.P' + l_2^! v_2.P')$. Independently of which branch is taken, the process proceeds as $P'$. Thus, if the broker is located in a process $Q$, then $P$ needs not be informed of the selected choice. The same is true for classical sessions where selection is a mixed-out choice of a single branch.

There are two other aspects that one should discuss when implementing mixed sessions on a message passing architecture other than the number of messages exchanged.

The first is related the type of broker used and to which values are revealed in a choice to the other party. In the case of the basic broker, only the chosen option value is revealed, and never to the broker itself. However, when we piggyback the values in the second type of broker, all values in the choice branches are revealed to the broker, even if they are not used in the end. This is even more striking in the case where one of the processes is the broker—the other party has access to all the possible values, independently of the choice that is taken.

The second aspect is also related to the values themselves which, in order to be presented in the choice, values must be computed *a priori*, even if they are not used in the choice.

When dealing with the privacy of the values, we can choose which type of broker to use depending on how much we want to reveal to the other party. However, to prevent computing before a branch is chosen, one should instead use classical sessions.

## 7 Related Work

*The origin of choice* Free (completely unrestricted) choice is central to process algebras, including BPA and CCS [3,26]. Here we usually find processes of the form $P + Q$, where $P$ and $Q$ are arbitrary process. Free choice is also present in the very first proposal of the $\pi$-calculus [30,31], even if Milner later uses guarded choice [28]. Sangiorgi and Walker's book builds on the pi-calculus with guarded (mixed) choice [38]. Guarded choices in all preceding proposals operate on possibly distinct channels—$x!\mathsf{true}.P + y?z.Q$— whereas choices on mixed sessions run on a common channel—$x(l!\mathsf{true}.P + m?y.Q)$. Kouzapas and Yoshida introduce the notion of mixed session in the context of multiparty session types [24]. Multiparty session types are projected into binary session types, hence the authors also consider mixed choices for binary sessions. This language is not as concise as the one we present, probably because it is designed so as to match projection from multiparty types.

Labelled-choices were embedded in the theory of session types by Honda et al. [18,19,41], where one finds primitives for value passing—$x!\mathsf{true}.P$ and $x?y.Q$—and, separately, for choice in the form of labelled selection—$x \triangleleft l.P$— and branching—$x \triangleright \{l_i \colon P_i\}_{i \in I}$—see Section 5. Coalescing label selection with output and branching with input was proposed by Vasconcelos [44] (and later used by Sangiorgi [37]) as a means to describe concurrent objects. Demangeon and Honda use a similar language to study embeddings of calculi for functions and for session-based communication [9]. All these languages offer only separated (unmixed) choices and only on the input side.

*Mixed choices in the Singularity operating system* Concrete syntax apart, the language of linear mixed choices is quite similar to that of channel contracts in Sing# [10]. Rather than explicit recursive types, Sing# contracts uses named states (akin to typestates [40]), providing for more legible contracts. In Sing#, each state in a contract corresponds to a mixed session $\mathsf{lin}\&\{l_i^\star S_i.T_i\}$ (contracts are always written from the consumer side) where each $l_i$ denotes a message tag, $\star$ the message direction (! or ?), $S_i$ the type of the value in the message, and $T_i$ the next state.

Stengel and Bultan showed that processes that follow Sing# contracts can engage in communication errors [39]. They further provide a realizability condition for contracts that essentially rules out mixed choices. Bono and Padovani present a calculus and a type system that models Sing# [6,7]. The type system ensures that well-typed processes are exempt from communication errors, but the language of types excludes mixed-choices. So it seems that Sing#-like languages only function properly under separated choice, yet our work survives under mixed choices. Contradiction? No! Sing# features asynchronous (or buffered) semantics whereas mixed sessions run under synchronous semantics. The operational semantics makes all the difference in this case.

*Synchronicity, asynchronicity, and choice* Pierce and Turner identified the problem: “In an asynchronous language guarded choice should be restricted still further since an asynchronous output in a choice is sensitive to buffering” [36] and Peters et al. state that “a discussion on synchrony versus asynchrony cannot be separated from a discussion on choice” [34,35]. Based on classical sessions, mixed sessions are naturally synchronous. The naive introduction of an asynchronous semantics would ruin the main results of the language (see Section 4). Asynchronous semantics are known to be compatible with classical sessions; see Honda et al. [20,21] for multiparty asynchronous session types and Fowler et al. [11] and Gay and Vasconcelos [16] for two examples of functional languages with session types and asynchronous semantics. So one can ask whether a language can be designed where mixed-choices are handled synchronously and separated-choices asynchronously, a type-guided operational semantics with by-default asynchronous semantics, reverting to a synchronous semantics when in presence of mixed-choices.

*Separation results* Palamidessi shows that the $\pi$-calculus with mixed choice is more expressive than its subset with separated choice [32]. Gorla provides a simpler proof [17] of the same result and Peters and Nestmann analyse the problem from the perspective of breaking initial symmetries in separated-choice processes [33]. Unlike the $\pi$-calculus with separated choices, mixed choices operate on the same channel and are guided by types. It would be interesting to look into separation results for classical sessions and mixed sessions. Are mixed sessions more expressive than classical session under some widely accepted criteria (those of Gorla [17], for example)?

*The origin of mixed sessions* Mixed sessions dawned on us when looking into an algorithm to decide the equivalence of context-free session types [1,42]. The algorithm translates types into (simple) context-free grammars. The decision procedure runs on arbitrary simple grammars: the right-hand sides of grammar productions may start with a label-output or a label-input pair for the same non-terminal symbol at the left of the production. We then decided to explore mixed sessions and picked the simplest possible language for the effect: the $\pi$-calculus. It would be interesting to look into mixed context-free session types, given that decidability of type equivalence is guaranteed.

## 8 Conclusion

We introduce mixed sessions: session types with mixed choice. Classical session types feature separated choice; in fact all the proposals in the literature we are aware of provide for choice on the input side only, even if we can easily think of choice on the output side. Mixed sessions increase flexibility in programming and are easily realisable in conventional message passing architectures.

Mixed choices come with a type system featuring subtyping. Typability is preserved by reduction. Furthermore well-typed programs are exempt from runtime errors. We provide suggestions on how to derive a type checking procedure, even if we do not formalise it. Classical session types are a particular case of mixed sessions: we provide for an encoding and show typing and operational correspondences.

We leave open the problem of looking into a *typed* separation result (or a proof of inseparability) between classical sessions and mixed sessions. An interesting avenue for further development includes looking for a hybrid type-guided semantics, asynchcronous by default, that reverts to synchronous when in presence of an output choice.

*Acknowledgements* We thank Simon Gay, Uwe Nestmann, Kirstin Peters, and Peter Thiemann for comments and discussions. This work was supported by FCT through the LASIGE Research Unit, ref. UIDB/00408/2020, and by Cost Action CA15123 EUTypes.
